# Diagnosis of Benign and Malignant Newly Developed Nodules on the Surgical Side After Breast Cancer Surgery Based on Machine Learning

**DOI:** 10.1155/tbj/8511049

**Published:** 2025-02-17

**Authors:** Zhixiang Wang, Qingqing Li, Yiran Wang, Linxue Qian, Xiangdong Hu, Dong Liu

**Affiliations:** ^1^Department of Ultrasound, Beijing Friendship Hospital, Capital Medical University, Beijing, China; ^2^Honors College, Nanjing Normal University, Nanjing 210023, China

**Keywords:** breast cancer, diagnostic modeling, machine learning, postoperative recurrence, ultrasound

## Abstract

**Objective:** To enhance the diagnostic accuracy of new nodules on the surgical side after breast cancer surgery using machine learning techniques and to explore the role of multifeature fusion.

**Methods:** Data from 137 breast cancer postoperative patients with new nodules from January 2016 to April 2024 were analyzed. Clinical, ultrasound, immunohistochemistry, and surgical features were combined. Multiple machine learning models, including support vector machine (SVM), random forest, gradient boosting, AdaBoost, and XGBoost, were trained and tested. Model performance was evaluated using stratified ten-fold cross-validation. Ablation experiments assessed the impact of different feature combinations on diagnostic performance.

**Results:** The SVM model performed best, with an AUC of 0.8664, an accuracy of 0.8099, a sensitivity of 0.565, and a specificity of 0.9267. Ablation experiments indicated that multifeature fusion significantly improved diagnostic performance, especially when combining clinical, ultrasound, immunohistochemistry, and surgical features. Gradient boosting and random forest models showed slightly inferior performance, while AdaBoost had balanced but lower effectiveness.

**Conclusion:** Machine learning, particularly the multifeature fusion SVM model, shows significant potential in diagnosing new nodules after breast cancer surgery. It can assist doctors in developing more effective treatment plans, improving patient outcomes. Future studies should expand sample sizes, include multicenter data, and explore advanced algorithms to further enhance diagnostic performance.

## 1. Introduction

Breast cancer remains a leading cause of morbidity and mortality among women worldwide. According to the World Health Organization, breast cancer has surpassed lung cancer to become the most frequently diagnosed cancer in women globally, with 2.3 million new cases each year, accounting for 11.7% of all cancer cases [1]. In China, breast cancer not only has the highest incidence rate among women but also ranks as a leading cause of cancer-related deaths among women under the age of 70 [2]. Despite advances in breast cancer treatment, postoperative recurrence and distant metastasis continue to pose significant challenges in clinical management [3].

The diagnosis of postoperative recurrent nodules remains a significant challenge in the follow-up of breast cancer. Newly developed nodules after breast cancer surgery may be benign postoperative reactions such as seromas [4], hematomas [5], or scar tissue [6], but they could also indicate malignant tumor recurrence [7]. The nature of these nodules is difficult to accurately differentiate using conventional ultrasound [8], leading to uncertainty in subsequent patient treatment. Existing follow-up methods, such as ultrasound, mammography, and magnetic resonance imaging (MRI) [9–11], each have their advantages but also certain limitations [12]. Ultrasound, due to its noninvasiveness, low cost, and convenience, is widely used in routine postoperative follow-up of breast cancer [13]. However, due to the overlapping ultrasound appearances of postoperative reactions and recurrent nodules [14], the accuracy (ACC) of ultrasound in distinguishing between benign and malignant newly developed nodules still needs improvement [15].

In recent years, machine learning technology has shown tremendous potential in medical imaging diagnosis [16]. By learning from large volumes of medical data, machine learning models can identify and classify complex imaging features, enhancing diagnostic ACC and efficiency [17, 18]. Existing studies have demonstrated that machine learning algorithms can achieve excellent results in the imaging diagnosis of various cancers, such as lung cancer [19] and brain tumors [20]. Especially for breast nodules, Zheng X et al. demonstrated that deep learning radiomics can be applied to predict axillary lymph node status in early-stage breast cancer. Meanwhile, Zheng et al. [27] utilized machine learning-based breast tumor ultrasound radiomics to preoperatively predict the axillary sentinel lymph node metastasis burden in early-stage invasive breast cancer [28]. However, research on the diagnosis of newly developed nodules in breast cancer using machine learning remains relatively limited.

This study aims to utilize machine learning technology, combined with conventional ultrasound and clinical indicators, to construct an efficient diagnostic model for newly developed nodules after breast cancer surgery. The study evaluates the application value of the machine learning model in distinguishing between benign and malignant newly developed nodules. In addition, it explores the diagnostic capabilities of different feature combinations, leveraging machine learning technology to identify more valuable diagnostic features.

## 2. Method

### 2.1. Data Collection

This study selected postoperative breast cancer patients who underwent routine ultrasound follow-up at the Beijing Friendship Hospital affiliated with Capital Medical University from January 2016 to April 2024. The subjects included patients who developed new local nodules in the surgical area and had obtained pathological results, totaling 137 cases. Inclusion criteria were newly developed local nodules after breast cancer surgery detected by ultrasound, clear pathological results for the new nodules, and informed consent from both the patients and their families. Exclusion criteria included poor quality of ultrasound images, absence of new nodules, presence of new nodules without pathological results, nodules not on the surgical side, new nodules in the surgical area accompanied by multiple organ metastases, incomplete clinical data, and severe diseases in other systems or major organs.

The color Doppler ultrasound diagnostic equipment used included Philips iu22/iUElite (Royal Philips, Netherlands), HI VISION Ascendus (Hitachi Aloka, Japan), and Mindray Resona R9 (Mindray Bio-Medical Electronics, Shenzhen), with probe frequencies of 5–14 MHz. The patients were examined in a supine or lateral position to fully expose the breast/chest wall, and a comprehensive scan of the breast area was performed. Ultrasound characteristics of the new nodules in the surgical area were recorded, including maximum diameter (cm), cystic-solid nature (purely solid, purely cystic, and cystic-solid mixed), echogenicity (hyperechoic, isoechoic, and hypoechoic), homogeneity (homogeneous/heterogeneous), margin (clear/unclear), shape (regular/irregular), posterior echo (enhanced/unchanged/attenuated), growth direction (horizontal/vertical), calcification (none/coarse/punctate), and blood flow signal (none/low/moderate/rich).

Blood flow signals were graded according to Alder's classification: Grade 0 indicated no blood flow signal within the lesion, Grade I indicated a small amount of blood flow signal with 1-2 punctate or rod-like blood flow signals, Grade II indicated a moderate amount of blood flow signal with 3-4 punctate or 1 longer vessel, and Grade III indicated abundant blood flow signals with ≥ 5 punctate or ≥ 2 longer vessels. Clinical data recorded for the enrolled subjects included gender, age at the time of new nodule development, preoperative pathological stage of breast cancer, type of surgery (total mastectomy/partial mastectomy), follow-up time at the appearance of new nodules (months), tumor markers at the time of nodule appearance, and pathological results of the new nodules.

### 2.2. Data Preprocessing, Model Construction, and Testing

To ensure data completeness and consistency, the following preprocessing steps were performed. First, the dates of breast cancer surgery and the appearance of new nodules were converted to a standard string format. Missing values were then filled in with “None” as the default value to avoid the impact of missing data on the analysis. In addition, all numerical columns were converted to string format to prevent the presence of nonnumeric characters in the data. The preprocessed data ensured high-quality input for subsequent modeling and analysis.

To evaluate the performance of different machine learning models in diagnosing newly developed nodules after breast cancer surgery, the study selected several commonly used machine learning models for comparison: random forest, support vector machine (SVM), gradient boosting, AdaBoost, and XGBoost [21–24]. These models were trained and tested on standardized data. Feature data were standardized using StandardScaler to improve model training performance and stability. The models were evaluated using stratified K-fold cross-validation [25], and the following evaluation metrics were computed: accuracy (ACC), area under the receiver operating characteristic curve (AUC), sensitivity (SEN), and specificity (SPE). For each cross-validation iteration, the model's prediction results were recorded, and confusion matrices were calculated to obtain ACC, SEN, and SPE. The receiver operating characteristic (ROC) curve and its AUC were used to assess the model's classification performance. Multiple cross-validations [26] for each model and feature combination provided average model performance to ensure the stability and reliability of the results.

### 2.3. Ablation Study

To further explore the impact of different feature combinations on the performance of diagnostic models, this study conducted an ablation study to assess the diagnostic efficacy of the following feature combinations: Clinical features, Ultrasound features, Immunohistochemistry features, Surgical features, Clinical features + Ultrasound features, Clinical features + Immunohistochemistry features, Clinical features + Surgical features, Ultrasound features + Immunohistochemistry features, Ultrasound features + Surgical features, Immunohistochemistry features + Surgical features, Clinical features + Ultrasound features + Immunohistochemistry features, Clinical features + Ultrasound features + Surgical features, Clinical features + Immunohistochemistry features + Surgical features, Ultrasound features + Immunohistochemistry features + Surgical features, and Clinical features + Ultrasound features + Immunohistochemistry features + Surgical features.

SVM was used as the core model to build and evaluate these feature combinations, and the performance metrics of ACC, AUC, SEN, and SPE were computed for each combination.

## 3. Result

### 3.1. Patient Information

The patient information is shown in Table 1.

### 3.2. Model Performance

**Figure 1:**
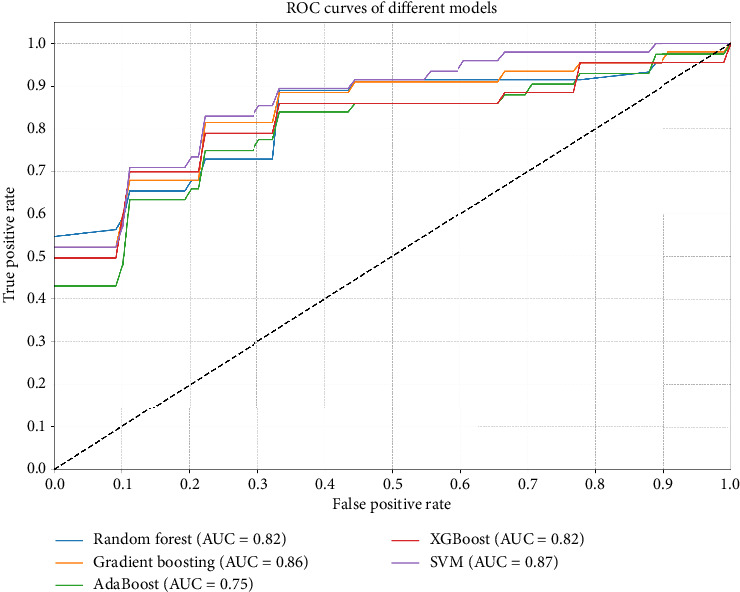
ROC curves of different models.

The study evaluated the performance of various machine learning models in diagnosing newly developed nodules after breast cancer surgery, with the results summarized in Figure 1 and Table 2. The SVM model demonstrated the best performance, with an AUC of 0.8664, ACC of 0.8099, SEN of 0.565, and SPE of 0.9267. The gradient boosting model also performed relatively well, with an AUC of 0.8533, ACC of 0.7659, SEN of 0.61, and SPE of 0.8389. The random forest model had an AUC of 0.8224, ACC of 0.7934, SEN of 0.57, and SPE of 0.9033. The XGBoost model showed balanced performance, with an AUC of 0.8189, ACC of 0.7791, SEN of 0.64, and SPE of 0.8478. The AdaBoost model performed slightly worse, with an AUC of 0.7506, ACC of 0.7225, SEN of 0.58, and SPE of 0.7844.

### 3.3. Ablation Study

**Figure 2:**
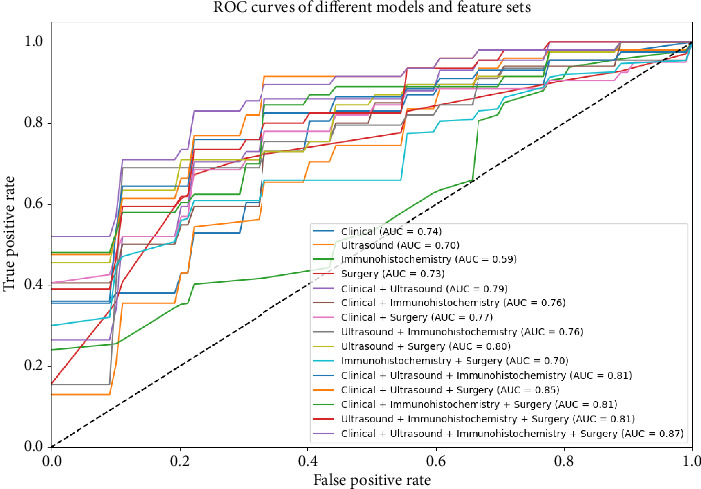
ROC curves of different models and feature sets.

The results of the ablation study are presented in Table 3 and Figure 2, showing the impact of different feature combinations on the performance of the logistic regression model. When using individual feature types—clinical features, ultrasound features, immunohistochemistry features, and surgical features—the model's performance was relatively weak. Specifically, with clinical features alone, the ACC was 0.7005, AUC was 0.7347, SEN was 0.2, and SPE was 0.9378. Using immunohistochemistry features alone resulted in the lowest SEN, at only 0.065, though the SPE reached 0.9778.

Combining multiple feature sets as input significantly improved model performance. Combining clinical and ultrasound features yielded an ACC of 0.7, AUC of 0.7894, SEN of 0.285, and SPE of 0.8933. Combining clinical, ultrasound, and surgical features resulted in better performance, with an ACC of 0.7742, AUC of 0.8478, SEN of 0.5, and SPE of 0.9044. The model performed best when all feature types were combined, achieving an ACC of 0.8099, AUC of 0.8664, SEN of 0.565, and SPE of 0.9267.

## 4. Discussion

Breast cancer remains the most common malignant tumor among women worldwide, and accurate diagnosis of postoperative recurrent nodules is crucial for improving patient prognosis. This study aimed to enhance the diagnostic ACC of newly developed nodules on the surgical side after breast cancer surgery using machine learning techniques. The data we utilized can mirror the current state of breast cancer comprehensively. It encompasses new trends in risk factors, treatment responses, and recurrence patterns. For example, novel treatment modalities and their associated side-effects that might impact the manifestation of postoperative nodules are more likely to be incorporated into our dataset. Given that alterations in treatment paradigms can lead to diverse presentations of recurrent nodules, both in terms of clinical manifestations and underlying biological characteristics, having such data is of great significance.

The results indicate that the SVM model performed the best when combining all features, achieving an AUC of 0.8664. This demonstrates that multifeature fusion plays a significant role in diagnosing postoperative recurrent nodules in breast cancer. By integrating clinical, ultrasound, immunohistochemistry, and surgical features, the model can capture more detailed information related to recurrence, thus improving diagnostic SEN and SPE. This multifeature fusion approach provides comprehensive information coverage, reducing bias from single features and enhancing model stability and ACC.

In contrast, while gradient boosting and random forest models showed good performance with certain feature combinations, their overall performance was slightly inferior to that of the SVM model. This may be due to these models' SEN to feature redundancy and correlations when handling high-dimensional data, leading to performance fluctuations. The AdaBoost model showed relatively balanced performance, but its overall effectiveness still fell short of the SVM.

From a theoretical perspective, the superior performance of SVM in diagnosing recurrent nodules in breast cancer can be attributed to specific reasons. First, SVM maximizes the margin between classes by finding the optimal hyperplane, which enhances the model's ability to generalize to unseen data and handle complex high-dimensional data. Second, SVM uses kernel functions to map the original feature space to higher-dimensional spaces, effectively capturing nonlinear relationships, which is crucial for dealing with complex interactions in medical data. In additionally, SVM is robust to feature redundancy and noise, maintaining good performance even with a large number of features. In contrast, random forest and gradient boosting models, although robust to some extent, are more susceptible to feature redundancy and noise, leading to unstable performance. KNN models, on the other hand, are highly dependent on the local structure of the data and are sensitive to uneven data distribution, resulting in lower SEN. Overall, SVM demonstrates significant advantages in handling high-dimensional, multifeature fused data for breast cancer postoperative recurrent nodule diagnosis, providing higher diagnostic ACC and stability.

The outstanding performance of the SVM model, which integrates clinical, ultrasound, immunohistochemistry, and surgical features, further emphasizes the significance of these features in diagnosing breast cancer postoperative recurrent nodules. Clinical features provide a comprehensive understanding of a patient's overall health and medical history. Ultrasound features can directly reflect the pathological characteristics of nodules. Immunohistochemistry features offer in-depth details about the biological behavior of the primary tumor, and surgical features supply crucial information regarding the surgical process and outcomes. The amalgamation of these diverse data sources enables a more accurate portrayal of the nodules, thus significantly enhancing the diagnostic ACC.

However, diagnosing postoperative breast nodules is an arduous task due to their complex nature. Postoperative changes such as scarring, inflammation, and tissue remodeling can closely imitate cancer recurrence. Benign conditions, for instance, fat necrosis, often exhibit similar imaging features to malignant nodules, frequently resulting in diagnostic errors. Clinicians currently face great challenges in accurately identifying recurrent nodules using existing diagnostic methods.

Adding to the complexity, breast cancer itself has highly variable biological behaviors across different subtypes. Some subtypes are more aggressive, making them relatively easier to detect, while others are more indolent and are extremely difficult to differentiate from normal tissue. This variability further exacerbates the challenge of achieving high SEN in the diagnosis of postoperative recurrent nodules.

In light of these challenges, the 0.565 SEN of our SVM model is in line with the difficulties commonly encountered in clinical practice. For the model to be immediately applicable in a clinical setting, an ideal SEN would be close to 1. Nevertheless, realistically, improving this metric necessitates a combination of refined diagnostic criteria, more advanced imaging techniques, and potentially new biomarkers. Despite the current SEN level, when the SVM model is used in conjunction with other clinical information and diagnostic tools, it can still play a substantial role in assisting clinicians in making well-informed decisions. It helps in shortlisting cases that require further investigation, thereby reducing unnecessary invasive procedures and enabling more targeted follow-up strategies.

Undoubtedly, machine learning models hold great promise in the diagnosis of breast cancer postoperative recurrent nodules. They can assist physicians in enhancing diagnostic ACC and reducing misdiagnosis rates. By improving diagnostic precision, these models contribute to the formulation of more effective treatment plans, ultimately improving patient prognosis. This not only increases survival rates but also elevates the quality of life by reducing unnecessary treatments and follow-up expenses. For example, accurately identifying malignant nodules can prevent treatment delays, and precisely detecting benign nodules can avoid unnecessary invasive examinations and surgeries.

## 5. Limitations and Future Work

This study has several limitations. First, it is a single-center research with a relatively small sample size, as the data are sourced from only one medical institution. This single-center characteristic severely restricts the generalizability of the model. Patient demographics, such as age, gender distribution, and underlying health conditions, can vary significantly across different centers. In the future, we plan to incorporate multicenter research to enhance the generalizability of our findings. This will allow us to collect a more diverse range of data, covering a broader spectrum of patient characteristics and healthcare settings, thus strengthening the validity and applicability of the model. Second, the sample size is relatively small, which may limit the generalizability of the model. Third, while the study incorporated multiple features, some potentially valuable factors, such as genetic information and patient lifestyle, were not included. These factors may have significant implications for recurrence risk. In addition, the study primarily employed traditional machine learning algorithms and did not fully utilize more advanced techniques like deep learning, which could limit the model's optimal.

Future research should focus on expanding the sample size and including data from multiple centers to enhance the model's generalizability and applicability. Multicenter datasets will allow the model to better accommodate different populations and medical practices, improving its widespread clinical utility and reliability. Moreover, exploring and integrating additional features such as genetic information, detailed medical history, and lifestyle factors could provide a more comprehensive diagnostic basis. This would help capture more potential feature associations, further enhancing diagnostic performance. In addition, future studies should incorporate advanced machine learning algorithms, such as deep learning, which excel in handling complex and high-dimensional data and can detect subtle features that traditional methods might miss. These improvements could extend the application of machine learning technology in clinical diagnosis of postoperative recurrent nodules in breast cancer, ultimately improving patient treatment outcomes and prognosis.

## 6. Conclusion

This study utilized machine learning techniques to enhance the diagnostic ACC of newly developed nodules after breast cancer surgery. The results indicated that a multifeature fusion logistic regression model performed the best, significantly improving diagnostic SEN and SPE. The SVM model demonstrated considerable potential in diagnosis, assisting physicians in developing more effective treatment plans and improving patient prognosis. Despite limitations such as small sample size and single-center data, future research should aim to increase sample size, incorporate multicenter data, and explore additional features and advanced algorithms to further improve diagnostic performance.

## Figures and Tables

**Table 1:** Comparison of ultrasound characteristics and clinical indicators between pathologically benign and malignant groups.

Clinical and ultrasonographic Indicators (*n* = 137)	Pathologically benign nodule (93)	Pathologic malignant nodules (44)	Statistic	*p*
Clinical indicators				
1. Age (means ± standard deviation)	59.95 ± 10.72	57.95 ± 13.79	*t*	0.401
2. Sex (m/f)	0/93	1/43	*χ* ^2^	0.701
3. Tumor-marker (normal/elevated)	77/16	33/11	*χ* ^2^	0.284
4. Surgery			*χ* ^2^	< 0.01
General cut	16 (17.2%)	26 (59.1%)		
Partial cut	77 (82.8%)	18 (40.9%)		
5. Preoperative staging(I/II/III/IV)	55/31/5/2	21/20/2/1	*χ* ^2^	0.593
6. Follow-up time (months, median).	21	52	Mann–Whitney U	< 0.01
Ultrasound indicators				
1. Maximum diameter line (cm, median)	1.4	1.6	Mann–Whitney U	0.027
2. Echoes			*χ* ^2^	0.206
High	2a (2.2%)	1a (2.3%)		
Equal	4a (4.3%)	0a (0.0%)		
Low	87a (93.5%)	43a (97.7%)		
3. Ingredient			*χ* ^2^	1.000
cystic solid	9 (9.7%)	4 (9.1%)		
Solid	84 (90.3%)	40 (90.9%)		
4. Homogeneity			*χ* ^2^	0.320
Homogenized	53 (57.0%)	29 (65.9%)		
Inhomogeneous	40 (43.0%)	15 (34.1%)		
5. Boundaries			*χ* ^2^	0.831
Clear	27 (29.0%)	12 (27.3%)		
Unclear	66 (71.0%)	32 (72.7%)		
6. Size			*χ* ^2^	0.127
Even	18 (19.4%)	4 (9.1%)		
Uneven	75 (80.6%)	40 (90.9%)		
7. Growth pattern			*χ* ^2^	0.826
Horizontal	79 (84.9%)	38 (86.4%)		
Vertical	14 (15.1%)	6 (13.6%)		
8. Rear echo			*χ* ^2^	< 0.01
Reinforce	10a (10.8%)	19a (43.2%)		
Invariably	25a (26.9%)	17a (38.6%)		
attenuation	58b (62.4%)	8b (18.2%)		
9. Blood flow			*χ* ^2^	< 0.01
None	71a (76.3%)	17a (38.6%)		
A smidgen	10a,b (10.8%)	9a,b (20.5%)		
Medium	10b (10.8%)	11b (25.0%)		
Enrichment	2b (2.2%)	7b (15.9%)		
10. Strong echo			*χ* ^2^	0.007
None	61a (65.6%)	33a (75.0%)		
Loud	12b (12.9%)	0b (0.0%)		
Punctiform	20a,b (21.5%)	11a,b (25.0%)		

*Note:* There is no difference between groups when the footnote a/b/(a, b) is the same, and there is a difference between groups when it is different.

**Table 2:** Different models' performances.

Model	ACC	AUC	SEN	SPE
Random forest	0.793406593	0.822361111	0.57	0.903333333
Gradient boosting	0.765934066	0.853333333	0.61	0.838888889
AdaBoost	0.722527473	0.750555556	0.58	0.784444444
XGBoost	0.779120879	0.818888889	0.64	0.847777778
SVM	0.80989011	0.866388889	0.565	0.926666667

**Table 3:** Performances of different models and feature sets.

Feature set	ACC	AUC	SEN	SPE
Clinical	0.700549451	0.734722222	0.2	0.937777778
Ultrasound	0.686813187	0.695555556	0.295	0.87
Immunohistochemistry	0.686263736	0.593194444	0.065	0.977777778
Surgery	0.751648352	0.732777778	0.6	0.828888889
Clinical + ultrasound	0.7	0.789444444	0.285	0.893333333
Clinical + immunohistochemistry	0.765384615	0.760555556	0.285	0.99
Clinical + surgery	0.744505495	0.764444444	0.57	0.827777778
Ultrasound + immunohistochemistry	0.700549451	0.753611111	0.2	0.935555556
Ultrasound + surgery	0.781318681	0.800555556	0.555	0.893333333
Immunohistochemistry + surgery	0.714285714	0.699027778	0.45	0.84
Clinical + ultrasound + immunohistochemistry	0.751098901	0.803611111	0.305	0.957777778
Clinical + ultrasound + surgery	0.774175824	0.847777778	0.5	0.904444444
Clinical + immunohistochemistry + surgery	0.77967033	0.804444444	0.54	0.892222222
Ultrasound + immunohistochemistry + surgery	0.773626374	0.809166667	0.44	0.935555556
Clinical + ultrasound + immunohistochemistry + surgery	0.80989011	0.866388889	0.565	0.926666667

## Data Availability

The datasets generated and/or analyzed during this study are not publicly available due to privacy and ethical considerations but can be obtained from the corresponding author upon reasonable request. All requests for data access will be reviewed by the author to ensure appropriate use. For more information on the data and how to request access, please contact the corresponding author.
